# *In vitro* neuronal network activity as a new functional diagnostic system to detect effects of Cerebrospinal fluid from autoimmune encephalitis patients

**DOI:** 10.1038/s41598-019-41849-z

**Published:** 2019-04-03

**Authors:** Henner Koch, Cristina E. Niturad, Stephan Theiss, Christian G. Bien, Christian Elger, Klaus-Peter Wandinger, Angela Vincent, Michael Malter, Peter Körtvelyessy, Holger Lerche, Marcel Dihné

**Affiliations:** 1 Department of Neurology and Epileptology, Hertie Institute of Clinical Brain Research, University of Tübingen, Tübingen, Germany; 2 Institute of Clinical Neuroscience and Medical Psychology, Medical Faculty, Heinrich Heine University, Düsseldorf, Germany; 3 Krankenhaus Mara, Epilepsy Center Bethel, Bielefeld, Germany; 4 Department of Epileptology, University Bonn Medical Center, Bonn, Germany; 5 Department of Neurology, University Hospital Schleswig-Holstein, Lübeck, Germany; 6 Institute of Clinical Chemistry, University Hospital Schleswig-Holstein, Lübeck, UK; 7 Neuroimmunology Group, Nuffield Department of Clinical Neurosciences, University of Oxford, West Wing, John Radcliffe Hospital, Oxford, OX3 9DU UK; 8 Department of Neurology, University Hospital, Cologne, Germany; 9 German Center for Neurodegenerative Diseases, University Hospital Magdeburg, Magdeburg, Germany

## Abstract

The intent of this study was to investigate if cerebrospinal fluid (CSF) from autoimmune encephalitis (AE) patients regulates *in vitro* neuronal network activity differentially to healthy human control CSF (hCSF). To this end, electrophysiological effects of CSF from AE patients or hCSF were measured by *in vitro* neuronal network activity (ivNNA) recorded with microelectrode arrays (MEA). CSF from patients with either N-methyl-D-aspartate-receptor-antibody (pCSF^NMDAR^, n = 7) or Leucine-rich-glioma-inactivated-1-Ab (pCSF^LGI1^, n = 6) associated AE suppressed global spiking activity of neuronal networks by a factor of 2.17 (*p* < 0.05) or 2.42 (*p* < 0.05) compared to hCSF. The former also suppressed synchronous network bursting by a factor of 1.93 (*p* < 0.05) in comparison to hCSF (n = 13). As a functional diagnostic test, this parameter reached a sensitivity of 86% for NMDAR-Ab- and 100% for LGI1-Ab-associated AE vs. hCSF at a specificity of 85%. To explore if modulation at the NMDAR influences effects of hCSF or pathological CSF, we applied the NMDAR-antagonist 2-Amino-5-phosphono-pentanoic acid (AP5). In CSF from NMDAR-Ab-associated AE patients, spike rate reduction by AP5 was more than 2-fold larger than in hCSF (*p* < 0.05), and network burst rate reduction more than 18-fold (*p* < 0.01). Recording ivNNA might help discriminating between functional effects of CSF from AE patients and hCSF, and thus could be used as a functional diagnostic test in AE. The pronounced suppression of ivNNA by CSF from NMDAR-Ab-associated AE patients and simultaneous antagonism at the NMDAR by AP5, particularly in burst activity, compared to hCSF plus AP5, confirms that the former contains additional ivNNA-suppressing factors.

## Introduction

Onconeuronal antibodies (Abs) against intracellular proteins like Hu, Ma or CV2 and those against cell-surface or soluble proteins like the NMDAR, LGI1 or contactin-associated-protein-like-2 (CASPR2) can be associated with AE^1^, and those against cell-surface proteins are thought to be causative. Limbic encephalitis, the most important clinical form of AE, is characterized by subacute (<3 month) alterations in mental status or psychiatric symptoms, seizures and typically magnetic-resonance imaging (MRI) findings with frequent but not inevitable inflammatory signs in CSF. Positive antibody testing can confirm the diagnosis. Therapy of AE consists of immunosuppression/-modulation with steroids, intravenous immunoglobulins, plasmapheresis or long-term regimes with rituximab or cyclophosphamide^2^.

However, the diagnosis of AE can be challenging, and a substantial portion (up to 44%) of patients are negative for known antibodies^3^. Moreover, clinical symptoms may be non-specific so that only the subacute course favors the diagnosis. Pathological results from MRI, electroencephalography and CSF are not AE specific, and autoimmune-Abs are sometimes clinically irrelevant, for instance in patients with small lung cancer and Hu-Abs without neurological disease^4^. Monitoring AE is important in order to schedule adequate duration and strength of immunosuppressive therapy, but accurate Ab-titers in serum and CSF are not always available and rarely correlate to clinical conditions^5^ to inform clinical decisions. Healthy human control CSF (hCSF) has been recently associated with increased neuronal activity and survival in several *in vitro* systems^6,7^.

**Figure 1:**
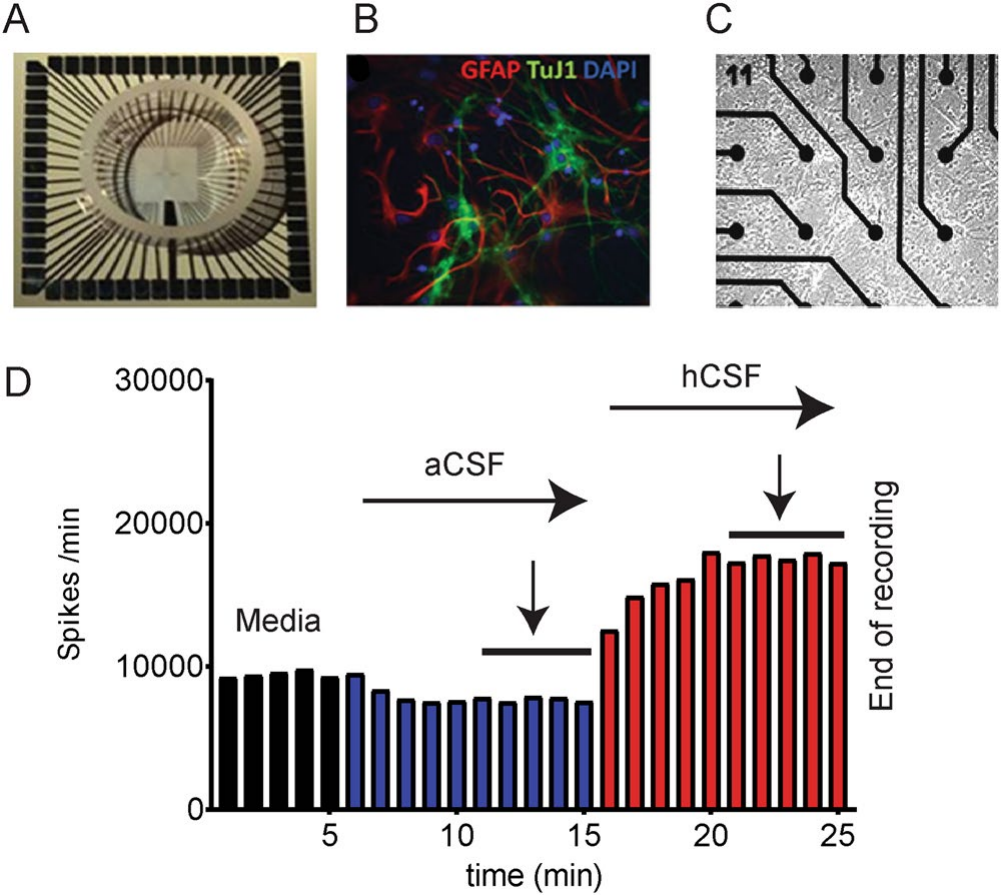
MEA System and experimental design. In (**A**) a whole MEA chip is depicted. In (**B**) a photomicrograph illustrates GFAP-positive (red) astrocytes and β−tubulin-positive (green) neurons. The blue color marks cell nuclei. (**C**) shows the neural population situated around some MEA electrodes. (**D**) Experimental design and exemplary spike rate response to exchange from culture media (black bars) to aCSF (blue bars) to hCSF (red bars). Measurements for quantification were taken at the last five minutes (marked by arrow) of aCSF and hCSF (of patients or healthy controls).

A functional read out system could be helpful in order to measure disease activity. In recent years, we could show that ivNNA measured by the MEA system (multiple extracellular electrodes that detect neuronal spiking activity, Fig. 1) is able to discriminate between hCSF or CSF from different central nervous system diseases^8,9^, including one case with NMDAR-Ab-associated encephalitis^10^. In the present study, we investigated, if ivNNA is able to discriminate between CSF from patients with AE associated with LGI1-Abs (n = 6) and hCSF (n = 13) and consolidate the data concerning AE associated with NMDAR-Abs (n = 7). Additionally, we investigated effects on ivNNA by a CASPR2-Ab CSF sample.

## Results

**Figure 2:**
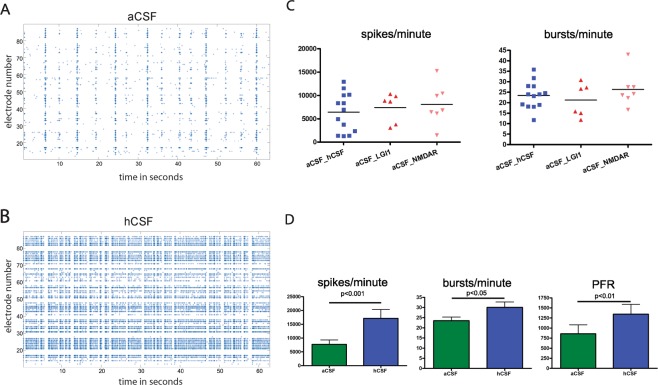
Human control CSF increases ivNNA in comparison to artificial CSF. (**A**,**B**) Shown are spike raster plots (SRPs) with time in seconds on the x-axis and electrode numbers (1–100) on the y-axis. Every single dot represents an extracellularly detected spike from neurons nearby individual electrodes. If spikes appear vertically aligned, spatially distributed neurons fire synchronized sequences of spikes termed network bursts, separated by quiescent periods. Between network bursts, activity gaps can be seen (inter burst intervals) with only some non-synchronized spikes. Note, SRPs under the influence of hCSF show more densely packed network bursts. (**C**) Baseline spike and burst rates recorded in aCSF prior to application of either hCSF, pCSF^LGI1^ or pCSF^NMDAR^ are shown: aCSF_LGI1, for instance, shows the baseline spike rate in aCSF before pCSF^LGI1^ was added. Only MEAs were used with a spike rate between 1,000 and 16,000 spikes/minute and a burst rate between 10 and 50 bursts/minute. The baseline spike rate per minute under aCSF of all 26 MEAs used for our experiments was between 1,287 and 15,236 (7,089 ± 766; 95% confidence interval 5,511–8,666). The burst rate per minute was between 12 and 43 (24 ± 1; 95% confidence interval 21–27). There was no significant difference between the baseline spike rates in the presence of aCSF used for later application of either hCSF, pCSF^LGI1^ or pCSF^NMDAR^ (**D**).

First, hCSF (n = 13) significantly increased absolute values of global spike activity, the number of network bursts, also illustrated in the spike raster plots (SRP), and the peak firing rate (PFR), compared to baseline activity under aCSF (Fig. 2). This phenomenon was seen in earlier studies^9^.

**Figure 3:**
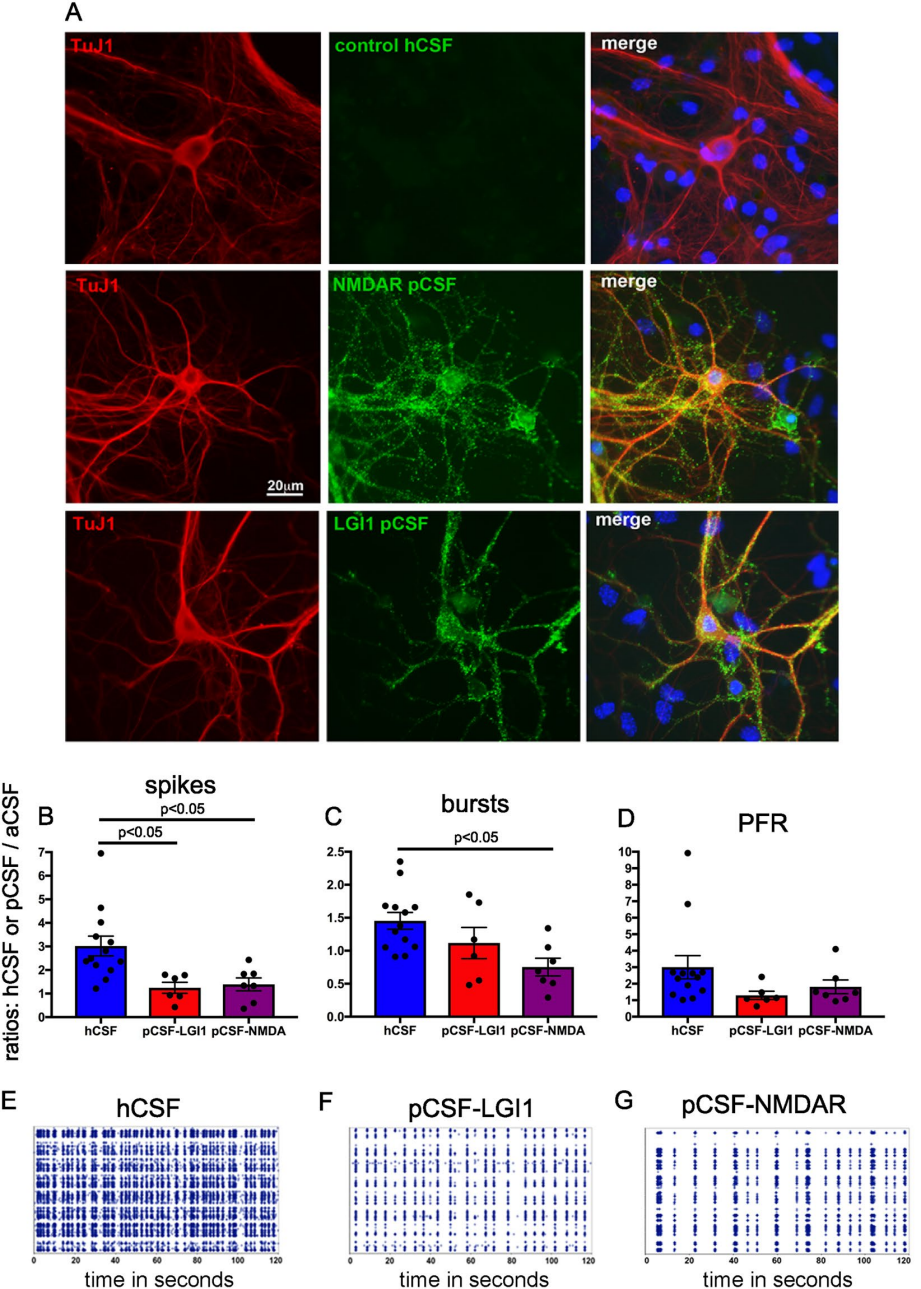
CSF from NMDAR-Ab encephalitis patients contains neuronal antibodies and does not increase  ivNNA in comparison to aCSF. (**A**) To verify the presence of neuronal antibodies in CSF from NMDAR-Ab or LGI1-Ab patients, we applied pCSF and hCSF samples on non-permeabilized dissociated primary mouse hippocampal neurons (10 days *in vitro*, βtubulin, TuJ1, immunocytochemistry, red) that are known to express NMDARs containing the target antigens. Indeed, pCSF^NMDAR^ and pCSF^LGI1^ showed a strong immunoreactivity (green) with overlap on neurons (merged photomicrograph, yellow), while control samples (hCSF) did not. In (**B**–**D**) ratios of absolute values for spikes/minute, bursts/minute and PFR  are given. We calculated the respective values for human CSF (either hCSF from controls, n = 13; pCSF^LGI1^, n = 6; or pCSF^NMDAR^, n = 7) divided by those for aCSF: pCSF-LGI1, for instance, shows the value pCSF^LGI1^/aCSF. Shown are all individual samples as dots, the mean value as bar graph, and error bars represent S.E.M. In (**E**–**G**) spike raster plots (SRPs) are illustrated, visualizing the decreased activity under the influence of pCSF^NMDAR^ (**G**) or pCSF^LGI1^ (**F**) compared to hCSF (**E**).

Using immunohistochemistry, we verified the presence of neuronal-antibodies in CSF samples from NMDAR- and LGI1-encephalitis patients. As illustrated in Fig. 3A, antibodies (diluted 1:10) in pCSF^NMDAR^ and pCSF^LGI1^, but not in hCSF, bind to β-tubulin+ mouse neurons.

To compare the abovementioned parameters between hCSF and pCSF samples, we normalized values by calculating ratios (hCSF or pCSF/aCSF). In the hCSF situation, ratios of spike rate, burst rate and the PFR were 3.02 (±0.42), 1.45 (±0.13) and 3.00 (±0.70), respectively. By contrast, in pCSF^NMDAR^, the ratio of the spike rate was only 1.39 (±0.28) and in pCSF^LGI1^ only 1.25 (±0.23, Fig. 3B–D). In pCSF^NMDAR^, the ratio of network bursts was only 0.79 (±0.13). These results represent a significantly reduced increase of activity in the presence of CSF from the patients compared to samples from healthy controls (p < 0.05, One Way ANOVA, Fig. 3B,C).

PFR ratios were not different between all groups. Representative SRPs illustrate network firing patterns from hCSF, pCSF^LGI1^ and pCSF^NMDAR^ (Fig. 3E–G). Interestingly, while the enhanced network activity observed after the application of hCSF returned back to the baseline levels after re-introducing aCSF, we did not detect a return to baseline levels after application of patient CSF (only tested for pCSF^NMDAR^).

To examine whether the differential effects of hCSF and pCSF^NMDAR^ on ivNNA could be NMDAR-mediated, we applied 50 µM of AP5 to hCSF and pCSF^NMDAR^.

**Figure 4:**
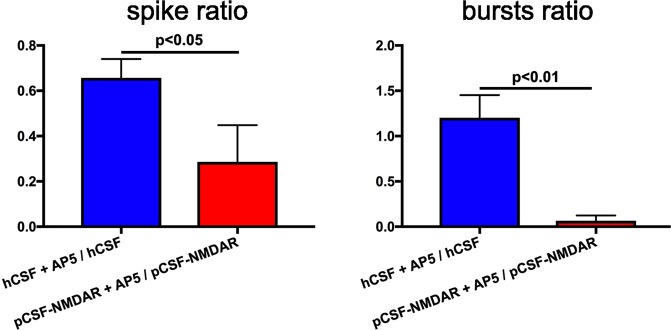
AP5 decreases network activity significantly more in pCSF^NMDAR^. In (**A**,**B**) effects of 50 μM AP5 on ivNNA under hCSF or pCSF^NMDAR^ influence are illustrated with the ratios given on the y-axis. Ratios of spike or burst activity after/before application of AP5 to hCSF or pCSF^NMDAR^ are given as illustrated on the x-axis. Note, AP5 reduced (below 1) spiking activity under hCSF and, even more, under pCSF^NMDAR^. However, the number of network bursts under hCSF remained nearly unchanged after AP5 application (around 1), with almost no remaining network bursts after AP5 application in pCSF^NMDAR^. For hCSF, we used 8 different CSF samples and performed 17 independent recordings in different MEA cultures. For pCSF^NMDAR^, we used 3 different CSF samples and performed 8 independent recordings. Error bars represent S.E.M.

AP5 strongly reduced spiking activity in pCSF^NMDAR^ even further, to 0.29 of initial level (±0.16, p < 0.05 rel. hCSF), while in hCSF AP5 only reduced activity to 0.66 (±0.08). Network bursting was even more prominently suppressed in pCSF^NMDAR^ to 0.07 of initial level (±0.06, p < 0.05), compared to hCSF at 1.2 (±0.25, Fig. 4A,B).

## Discussion

Antibodies to neuronal surface proteins now play an important role in the diagnosis of subacute forms of encephalitis, but not all patients with clinically-defined autoimmune encephalitis have positive antibodies and the acute functional effects have not been examined. Here we show that ivNNA of primary neuronal mouse cultures measured by MEA is suppressed by pCSF^NMDAR^ and pCSF^LGI1^ in comparison to hCSF. Interestingly, hCSF stimulated global spiking activity and network bursting compared to aCSF that only contains ions and glucose. The effect of hCSF has been reported previously although the reasons for the phenomenon are unknown. Possibly there are additional ivNNA-supporting factors in healthy control hCSF^11^. However, pCSF^NMDAR^ did not significantly change ivNNA compared to aCSF, thus suppressed electrophysiological parameters compared to hCSF. In pCSF^NMDAR^, a possible mechanism could be modulation at the NMDAR itself which is known to play a pivotal role in ivNNA^9^ and may be also important in the pathophysiology of NMDAR-Ab-associated encephalitis. Control hCSF seems to constitute a physiological environment supporting ivNNA and NMDAR-blocking by AP5 suppressed ivNNA parameters. When AP5 was used to block NMDAR in pCSF^NMDAR^, spiking and bursting activity was further suppressed supporting the idea that NMDAR-Ab and AP5 may work synergistically. Interestingly, effects of pCSF^NMDAR^ are rapid within minutes, arguing for the hypothesis that effects of pCSF^NMDAR^ on ivNNA might be directly receptor-mediated^12^, since the already described binding and internalization of the antibody-receptor complex are too slow to be responsible for the observed effects here. However, in a previous study using patch clamp recordings, direct short-term effects within 30 minutes of NMDAR antibodies on NMDAR-mediated currents were determined and no significant effect was observed^13^. Other patch clamp studies, that investigated long-term effects (18–24 hours incubation) of NMDAR antibodies found reduced NMDAR activity (reduced NMDAR currents)^14,15^. As long-term effects might be related to NMDAR internalization, it remains unclear if NMDAR antibodies exert direct electrophysiological effects. Our study supports the possibility of direct and rapid effects. Direct receptor-mediated effects are further supported by our experiments with AP5, that indicate a stronger dependence of ivNNA on NMDAR function under the influence of pCSF^NMDAR^ in comparison to hCSF. Since NMDAR antibodies have been reported to lead to prolonged open states of NMDAR^16^ this effect would then be specifically mediated by the NR1 subunit of the NMDAR, which is known to be the target of antibodies in NMDAR encephalitis patients^14^. However, it could also be postulated that the NMDAR antibodies lead to a reconfiguration of the network function that is consequently more drastically modified by blocking NMDAR which is described in other networks^17^.

pCSF^LGI1^ also suppressed ivNNA. Again, the rapid effects reported here and the role of LGI1-Ab on voltage-gated potassium channel complexes suggest direct pCSF^LGI1^-Ab-related mechanisms. Interestingly, under pCSF^LGI1^, only global spiking activity was suppressed, while network bursting was preserved, indicating a different mechanism in comparison to pCSF^NMDAR^.

Because of different effects in pCSF^NMDAR^ and pCSF^LGI1^, we do not assume that additional factors in pCSF, such as drugs administered to the patients, play a major role for the observed effects. We would then also expect a higher variability between patients, since they presumably received different drugs at distinct dosages. The clinical data at the time of CSF sampling were not systematically available and drug levels were not measured, so that we could not correlate drugs that were applied to patients to measured effects on ivNNA. Nevertheless, we cannot exclude such drug effects contaminating our results.

From receiver-operating characteristic (ROC), we derived a tentative spike ratio threshold (rel. to aCSF) below 1.9 for discriminating pCSF against hCSF. With this setting, we obtained a specificity of 85% for both at a sensitivity of 86% in NMDAR-Ab-associated encephalitis patients (normal vs. anti-NDMAR-encephalitis) or 100% in LGI1-receptor-encephalitis patients. If this threshold was applied, an additional CASPR2-Ab sample (spike ratio 0.56) that was also measured on MEAs for the actual study lay in the pathological range. As CASPR2 is essential for correct localization of voltage-gated potassium channels in the central nervous system^18^, direct effects of CASPR2-Ab on ivNNA are conceivable. Thus, functional CSF diagnostics with ivNNA appears to be sensitive to AE CSF samples from a spectrum of individual patients.

In summary, we could demonstrate an overall correlation between ivNNA and AE diagnosis. Like in traumatic-brain-injury (TBI) and Dementia-with-Lewy-Bodies (DLB)^8,9^, also in AE, application of ivNNA can image functional effects of pathological CSF. In those diseases, pathological CSF led to diversely regulated ivNNA. For instance, in AE, network bursting was reduced but preserved while it was nearly abolished in TBI. In DLB, spiking activity was reduced, while it remained unchanged in CSF samples from Parkinson´s disease patients. In NMDAR-Ab-associated encephalitis, many previous reports have shown that NMDAR-Ab cause loss of NMDARs and result in reduced NMDAR function that appears within days^15^. Suppression of ivNNA, however, is rapid within minutes, and thus, possibly related to additional mechanisms that are directly receptor-mediated. In TBI, NMDAR-modulating substances like small amino acids are elevated in CSF and may cause rapid ivNNA suppression. Our LGI1-Ab data also provide electrophysiological evidence, demonstrating a direct effect of pCSF^LGI1^ on functional neuronal networks.

In TBI patients, we were able to demonstrate quantitative correlations of ivNNA to a clinical score, the Glasgow-Coma-Scale^9^. To show similar correlations of clinical conditions and ivNNA for NMDAR-Ab-associated encephalitis patients, further investigations of cognitive states at the time of taking CSF and its effects on ivNNA are necessary. Such effects could be shown during the course of the disease for a single patient with NMDAR-Ab-associated AE^10^. As a perspective, functional CSF diagnostics could be developed further to help monitor AE response to treatments, and to identify patients with novel antibodies.

## Methods

All experimental methods were carried out in accordance with relevant guidelines and regulations and were approved by an institutional committee. Experiments were approved by the local Animal Care and Use Committee (Regierungspraesidium Tuebingen, Tuebingen, Germany).

### Cell preparation

We plated dissociated hippocampal neural cultures prepared from E17 mice on microelectrode arrays (MEAs, Fig. 1A) as described in Hedrich and colleagues^19^. Briefly, pregnant females were killed using CO_2_, and embryos were quickly taken out and decapitated. By microsurgical dissection, hippocampi were isolated and washed three times with 4 °C magnesium- and calcium-free HBSS (PAA Laboratories GmbH) before treatment for 15 minutes with 2.5% trypsin. Subsequently, tissues were rinsed in DMEM with fetal bovine serum (Biochrom AG), L-glutamine (Invitrogen) and penicillin/streptomycin (Invitrogen) to block the trypsin reaction. 150,000 cells in 110 µl of solution were plated on MEAs coated with poly-D-lysine solution (5 mg poly-D-lysine in 100 ml of HBSS, filtered with a 0.45 µm filter) and 500 µl of DMEM with fetal bovine serum (Biochrom AG), L-glutamine (Invitrogen) and penicillin/streptomycin (Invitrogen). After cells had settled down for 4 h, MEAs were flooded with Neurobasal culture medium (Invitrogen) supplemented with B27 (Invitrogen), glutamine, and penicillin/streptomycin.

### MEAs

MEAs (Multichannel Systems) had a square grid of 60 planar Ti/TiN electrodes of 30 µm diameter and 200 µm spacing. Electrodes had an input impedance of 30–50 kΩ according to the specifications of the manufacturer. Signals from all 60 electrodes were simultaneously sampled at 25 kHz, visualized, and stored using the standard software MC_Rack provided by Multi Channel Systems. Spike detection was performed off-line by the SPANNER software suite (RESULT Medical). The networks show sparse or coordinated spike activity; the latter is defined as rhythmic spiking across multiple spatially distributed extracellular electrodes leading to synchronous network bursts (cf. Theiss *et al*.^8^). Number of spikes per minute was calculated as global measure of action potential activity in the entire network. Coordinated firing across the entire network was quantified by the number of network bursts per minute.

Network burst detection was performed in a three-step procedure: First, spikes detected on all electrodes were aggregated in consecutive non-overlapping 5 ms bins. Second, this raw network firing rate (with 5 ms temporal resolution) was smoothed using a normalized Gaussian kernel with standard deviation 100 ms. Network burst onset and termination were determined when this 100 ms smoothed network firing rate exceeded the slowly varying 1-second moving average, using the actual spike time stamps of the first and last spike in the time interval considered. Consecutive network bursts were merged into a single burst, if firing rate valleys between bursts were too shallow, or if bursts were less than 200 ms apart. Finally, the peak firing rate (PFR) of a given network burst was defined as the maximum of the 100 ms smoothed network firing rate within this burst. Very low-firing bursts were discarded, if their PFR did not exceed 10% of the average of the top five peaks, or if less than four electrodes contributed. Network burst detection was eventually checked by visual inspection. In this way, synchronous network activity on time scales of tens to several hundreds of milliseconds could reliably be captured^19^ and network bursts represent a measure of synchronous network activity.

### Immunocytochemistry

For immunocytochemical investigations of cell composition from E17 mice hippocampi on MEAs, cultured cells were washed in phosphate-buffered saline (PBS) and fixed for 15 min in 4% paraformaldehyde. Primary antibodies were monoclonal mouse antibodies to β-tubulin III (1:750, TuJ1, Sigma) and *glial fibrillary acidic protein* (GFAP 1:1000, Chemicon). Appropriate secondary antibodies coupled to Cy2 or Cy3 (1:750, Dianova, Hamburg, Germany) were applied. Cell cultures were counterstained for one minute with 4′,6-Diaminodino-2-Phenylindol (DAPI, 2 μg/ml, Serva) to visualize cell nuclei. To verify the presence of neuronal antibodies in pCSF^NMDAR^ and pCSF^LGI1^, we applied pure pCSF on β-tubulin III-positive (Cy3) mouse hippocampal neurons (E17, 10 days *in vitro*) and detected human antibodies by anti-human secondary antibodies (Cy2, 1:500, abcam, Cambridge, UK).

Images were collected with a confocal-laser scanning microscope (LSM 510 Zeiss, Germany). Cultures are illustrated in Fig. 1B,C. *In vitro* NNA develops by multiple interconnected neuronal sub-types and astrocytes. After a 3-week maturation period cultures were used for experiments.

### CSF collection and patient collective

The collection and use of CSF for this study was approved by the Ethics committee of the university Tübingen (Eberhard-Karls-Universität, 663/2012BO2). Samples were collected by lumbar puncture during diagnostic work up. The use of external CSF samples for research purposes was approved by local internal review boards and informed consent was obtained from all subjects. In particular, we withdrew at least 12 ml, centrifuged the CSF at 4000 g for 10 minutes at 4 °C. 100 µl aliquots were frozen at −80 °C within 2 hours. For each experiment we used one 100 µl aliquot and never reused, refroze or rethawed CSF samples after application to MEAs. Healthy human control CSF (hCSF) was taken from healthy individuals that underwent lumbar puncture to exclude acute neurological diseases and had normal CSF results concerning cell-count, total protein, glucose and lactate concentrations (13 control samples: 9F (69%), 4M; average age: 50.5 years). All patients with limbic encephalitis were diagnosed according to typical clinical signs and symptoms^1^ and including antibody positivity of NMDAR-Ab or LGI1-Ab in their CSF and serum (7 NMDAR samples: 6F (86%), 1M; average age: 49.3 years; 6 LGI1 samples: 2F (33%), 4M; average age: 53.2 years). Some of those patient CSF samples showed a slight inflammatory syndrome with slight pleocytosis and slightly elevated total protein. Specific antibody testing was performed by indirect immunofluorescence tests with primary antibodies from patients biofluids and a commercial second IgG-Fc-region binding fluorescence antibody. These routine procedures were performed with commercially available specific transfected HEK cells (EUROIMMUN, Lübeck,Germany) or live cell-based assays (Oxford) for each antibody, respectively.

### Experimental protocol of MEA recordings under CSF

The detailed experimental design can be found elsewhere^8^. Briefly, MEAs with cultures aged 21–28 DIV were transferred from the incubator to the amplifier headstage, networks were covered with a Potter cap to prevent evaporation and keep osmolarity constant and were left to equilibrate for 10–20 minutes, controlled by visual inspection. Each experiment then lasted 25 minutes, starting with a 5-minute recording in culture medium, followed by two 5-minute recordings of baseline activity in artificial CSF (aCSF: 150 mM sodium, 1 mM calcium, 3 mM potassium, 1 mM magnesium, 10 mM HEPES hemisodium salt, 10 mM glucose), and immediately followed by two 5-minute recordings in either human CSF (hCSF, healthy controls) or patient CSF (pCSF, AE patients) after complete liquid exchange. We always performed a full medium exchange by gentle pipetting to avoid shear strain on the networks. MEA recordings were interrupted before medium exchange and restarted shortly afterwards, so that no pipetting artifacts entered into the analysis. After exchanging either culture medium to aCSF, or aCSF to human control/patient CSF, we observed network equilibration during the first 5 minutes and checked stable activity levels during minutes 6 to 10. Only stable activity from minutes 6 to 10 was used for further analysis, and ratios “(activity under pCSF)/(activity under aCSF)” were calculated for each individual MEA (Fig. 1D). All CSF samples were buffered to pH 7.4 by adding 1 µl HEPES hemisodium salt from a 1M stock solution to 100 µl CSF, which barely changed liquid volume (by 1%) or osmolarity (by 3%). We performed all recordings while networks were covered with Potter caps to prevent evaporation and to keep osmolarity constant. In some experiments, the NMDA receptor antagonist AP5 (50 µM) was directly added to MEAs exposed to hCSF or pCSF^NMDAR^.

To ensure homogeneous baseline activity in aCSF, adjusted to pH 7.4 with HEPES hemisodium salt, we only included MEAs whose activity lay within predefined ranges (Fig. 2C). We then applied one sample of either hCSF or pCSF, also adjusted to pH 7.4, and normalized their activity to the mean of that sample in the preceding aCSF recording. We confirmed pH stability during the time course of the experiment by additional pH measurements at the end of the recording. For all groups, we additionally measured sample osmolarity and the ion concentrations of sodium, potassium, calcium and magnesium in a subsample of patients’ CSF. In these, there were no statistically significant differences between the groups for any of these parameters, as well as between different ages.

### Statistics

Each CSF sample was tested on ≥2 independent MEAs, and the means of the pooled results for individual patients’ CSFs were compared between the patient and control groups. Statistical analyses were performed with Graph Pad Prism 7 using paired t-test or ANOVA with Bonferroni correction for multiple comparisons. Means ± standard error of the means (SEM) are presented in text and figures.

### Receiver-operating characteristic

For a binary CSF classification as either “control” or “NMDAR/LGI-1 patient” CSF, sensitivity was defined as the proportion of true control CSF samples that had been correctly classified as controls: Sens = TP/(TP + FN), and classification specificity as the proportion of true patient CSF samples that had been correctly classified as patient CSF: Spec = TN/(TN + FP). Here, true positive (TP) denotes true control CSF correctly classified as control CSF, true negative (TN) denotes true patient CSF correctly classified as patient CSF, while false positive (FP) denotes true patient CSF falsely classified as control CSF and false negative (FN) denotes true control CSF falsely classified as patient CSF. Varying the threshold between “predicted control CSF” and “predicted patient CSF” in the change of spike activity level, i.e. the ratio (# spikes/min under h- or pCSF)/(# spikes/min under aCSF), the receiver-operating characteristic (ROC) curve was obtained by plotting sensitivity vs. 1-specificity. From this curve, an optimal classification threshold was obtained by maximizing Youden’s index (=sensitivity + specificity − 1), yielding a spike activity ratio threshold of 1.9 for discriminating between control and patient CSF.
